# Transcription Analysis of the Porcine Alveolar Macrophage Response to *Mycoplasma hyopneumoniae*

**DOI:** 10.1371/journal.pone.0101968

**Published:** 2014-08-06

**Authors:** Li Bin, Du Luping, Sun Bing, Yu Zhengyu, Liu Maojun, Feng Zhixin, Wei Yanna, Wang Haiyan, Shao Guoqing, He Kongwang

**Affiliations:** 1 Institute of Veterinary Medicine, Jiangsu Academy of Agricultural Sciences, Key Laboratory of Veterinary Biological Engineering and Technology, Ministry of Agriculture, Nanjing, Jiangsu Province, China; 2 Jiangsu Co-innovation Center for Prevention and Control of Important Animal Infectious Diseases and Zoonoses, Yangzhou, China; Cornell University, United States of America

## Abstract

*Mycoplasma hyopneumoniae* is considered the major causative agent of porcine respiratory disease complex, occurs worldwide and causes major economic losses to the pig industry. To gain more insights into the pathogenesis of this organism, the high throughput cDNA microarray assays were employed to evaluate host responses of porcine alveolar macrophages to *M. hyopneumoniae* infection. A total of 1033 and 1235 differentially expressed genes were identified in porcine alveolar macrophages in responses to exposure to *M. hyopneumoniae* at 6 and 15 hours post infection, respectively. The differentially expressed genes were involved in many vital functional classes, including inflammatory response, immune response, apoptosis, cell adhesion, defense response, signal transduction, protein folding, protein ubiquitination and so on. The pathway analysis demonstrated that the most significant pathways were the chemokine signaling pathway, Toll-like receptor signaling pathway, RIG-I-like receptor signaling pathway, nucleotide-binding oligomerization domains (Nod)-like receptor signaling pathway and apoptosis signaling pathway. The reliability of the data obtained from the microarray was verified by performing quantitative real-time PCR. The expression kinetics of chemokines was further analyzed. The present study is the first to document the response of porcine alveolar macrophages to *M. hyopneumoniae* infection. The data further developed our understanding of the molecular pathogenesis of *M. hyopneumoniae*.

## Introduction

*Mycoplasma hyopneumoniae* is the etiological agent of swine enzootic pneumonia, a chronic nonfatal disease affecting pigs of all ages. It is characterised by high morbidity and low mortality, resulting in significant economic losses due to decreased performance of pigs and the cost of medication [1]. *M. hyopneumoniae* predisposes animals to concurrent infections with other respiratory pathogens including bacteria, parasites and viruses. *M. hyopneumoniae* is also considered to be one of the primary agents involved in the porcine respiratory disease complex (PRDC) [2].

*M. hyopneumoniae* has been found to attach to the cilia of epithelial cells in the lungs of swine. They cause cilia to stop beating (ciliostasis), clumping and loss of cilia, eventually leading to epithelial cell death, which is the source of the lesions found in the lungs of pigs with porcine enzootic pneumonia [3]. On a cellular level, mononuclear cell infiltration of peribronchiolar and perivascular areas occurs. Then, *M. hyopneumoniae* actively suppresses immune systems of the host during early stages of pneumonia by inhibiting macrophage-mediated phagocytosis. The response of the host immune system causes the lesions seen in the lung tissue of infected swine by increasing phagocytic and cytotoxic activities of macrophages and initiating the chronic inflammatory response [4]. Increased production of proinflammatory cytokines, including interleukin (IL)-1β, tumor necrosis factor (TNF)-α, IL-6, IL-8 and IL-18 in the *M. hyopneumoniae* infected host also leads to a greater recruitment of neutrophils [4]–[9]. However, the factors involved in promoting protective immunity and/or the inflammatory responses against *M. hyopneumoniae* are not fully understood, and the cellular sensors and signaling pathway involved in these process has not yet been elucidated.

Innate immunity is the first line of defense for host protection against invading pathogens. Pattern recognition receptors (PRRs) are expressed in cells of the innate immune system, it include Toll-like receptors (TLR), RIG-I-like receptors (RLR) and NOD-like receptors (NLR). Pathogen-associated molecular patterns (PAMPs), derived from different pathogens, are recognized by PRRs, resulting in the release of inflammatory cytokines and interferons, and the boosting of host defenses. As a major component of the host innate immunity, macrophages have essential roles in host defense to infection [10], [11]. Host-pathogen interactions during *M. hyopneumoniae* infection are complicated, the interactions between *M. hyopneumoniae* with porcine alveolar macrophages have been less studied [7], [8], but the detailed mechanisms of how porcine alveolar macrophages response to *M. hyopneumoniae* infection are not well elucidated. To study the molecular mechanisms underlying the host response to pathogenic microorganisms in macrophages, microarrays have been widely used in recent years [12]–[15]. In the current study, we applied this high throughput cDNA microarray assay to improve our understanding of the innate immune response of macrophages to *M. hyopneumoniae* infection.

## Results

### Gene expression analysis during *M. hyopneumoniae* infection

**Figure 1:**
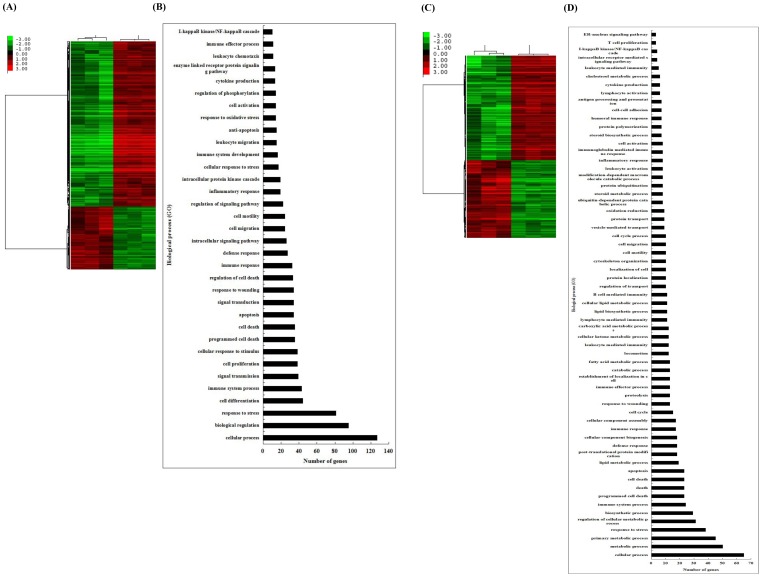
Clustering and characterization of the differential expression of genes. (A) The DE genes showing clear functional annotation at 6 HPI have been selected for cluster analysis which is described in methods. Each row represents a separate gene, and each column represents a experiment sample. Color legend is on the left, the color scale ranges from saturated green for log ratios −3.0 and above to saturated red for log ratios 3.0 and above. Red indicates increased gene expression levels; green indicates decreased levels compared with normal samples. (B) Categories of annotated genes based on biological process GO term at 6 HPI. (C) The genes showing clear functional annotation at 15 HPI have been selected for cluster analysis. (D) Categories of annotated genes based on biological process GO term at 15 HPI.

To investigate the pathogenesis of *M. hyopneumoniae*, the differential gene (DE) expression profile of PAM, after infection with *M. hyopneumoniae* was determined. Genes whose relative transcription levels showed a fold change (FC)≥2 (*p*≤0.05) were considered to be up-regulated, and those with a FC≤0.5 (*p*≤0.05) were considered to be down-regulated. Genes whose relative transcription levels had a FC greater than 0.5 or less than 2 were considered to have no notable change in expression levels. In this study, 1033 and 1235 DE genes were detected for active infection with *M. hyopneumoniae* compared with the control group at 6 and 15 hpi, respectively, *p*≤0.05 (Figure 1A and 1C). At 6 hpi, 747 genes were up-regulated and 286 down-regulated, whilst at 15 hpi 706 genes were up-regulated and 529 down-regulated.

### qRT-PCR validated DE genes

Microarray experiments yield a large amount of data, therefore it is important to validate differential expression by independent methods. To confirm the statistical significance of our findings, we performed quantitative real-time PCR (qRT-PCR) analysis on the relevant genes from our original samples used in the microarray study. Five up-regulated and five down-regulated genes, were selected for qRT-PCR analysis. Table 1 compares the results obtained from the microarray analysis with those of qRT-PCR. The results demonstrated that changes in expression levels of the ten selected genes as detected by qRT-PCR, were consistent with the changes (up-regulation or down-regulation) predicted by microarray analysis. However, the FCs differed markedly between microarray and qRT-PCR. This discrepancy may be attributable to technical differences encountered in the methods of analysis and normalization. Alternatively, the data could indicate that results from the microarray analysis are good indicators of overall changes in gene expression.

**Table 1:** Validation of microarray results by qRT-PCR.

GenBank ID	Gene symbol	Primers sequence (5′-3′)	Regulation	Microarray FC*	Real-time PCR FC
NM_214055	IL1β	F:GTGATGCCAACGTGCAGTCT	up	41.07	56.25
		R:TGGGCCAGCCAGCACTAG			
NM_213867	IL8	F:AGTTTTCCTGCTTTCTGCAGCT	up	4.16	8.26
		R:TGGCATCGAAGTTCTGCACT			
NM_214194	CD40	F:AGAACCACCCACTTCATGCAA	up	2.08	4.23
		R:TGGCGGGCACAAATTACA			
NM_001164511	BCL2A1	F:TTTGCATTTGAAGGTATTCTCATGA	up	4.15	8.95
		R:AATCTCCTTGTACGTGTCCACATC			
NM_214022	TNF-α	F:CGTTGTAGCCAATGTCAAAGCC	up	6.88	10.79
		R:TGCCCAGATTCAGCAAAGTCCA			
NM_001110425	CD302	F:TGGTTTCACCACAGTTTTCTCAA	down	2.48	3.25
		R:CCTCTGCAACTACCAAAACACAAT			
NM_001206441	TAP2	F:GGACTCTTCGGCTTCATGCT	up	2.16	2.03
		R:CGCTATCGTGAGAGGCATCTG			
NM_001113707	SLA	F:CACACACACCCAACCCTTCTG	down	3.01	2.85
		R:TGGTTTTGGCCACTTGCA			
NM_214162	CASP1	F:GCCAAGAGGGAGCCTCAAG	down	2.1	6.71
		R:CTCTGCTGACTTTTCTTTCCATAGC			
NM_001204769	NLRX1	F:TCTGCTGCGCAAATACATGTT	down	2.11	5.32
		R: CCATAGCGGCCCACATACTT			
NM_213779	CCL4	F: GCAAGACCATGAAGCTCTGC			
		R: AAGCTTCCGCACGGTGTATG			
NM_001164515	CCL8	F:AAGACCAAAGCCGACAAGGA			
		R: TCATGGAATTCTGGACCCACT			
NM_001001861	CXCL2	F: CCGTGCAAGGAATTCACCTC			
		R: TGCGGGGTTGAGACAAACTT			
NM_001008691	CXCL10	F: CCCACATGTTGAGATCATTGC			
		R CATCCTTATCAGTAGTGCCG			
	GAPDH	F:ACATGGCCTCCAAGGAGTAAGA			
		R:GATCGAGTTGGGGCTGTGACT			

*Fold change.

### Analysis of DE genes by gene ontology (GO)

All DE genes were annotated on the basis of the gene ontology (GO) database using Visualization and Integrated Discovery (DAVID). At 6 hpi, the DE genes mainly clustered into functional groups: inflammatory response (e.g. IL-1β, CCL4, CXCL10, CD14, TNF-α), immune response (e.g. TLR2, NFKBIA, IL7R, IL-8), apoptosis and anti-apoptosis (e.g. CASP10, BCL2A1, PIK3R1, NFKBIA, BTG2, PSEN1), programmed cell death (e.g. CXCR4, SOD2, NFKB1, PRK), defense response (e.g. TLR2, S100A8, CCL3L1, CD40), signal transmission, signal transduction cytokine production, I-kappaB kinase/NF-κB cascade and so on (Figure 1B).

Of interest, was the over-expression of up-regulated genes associated with immune responses and inflammatory responses, suggesting an important role for these genes in *M. hyopneumoniae* infections (Table 2). Of these genes, 20 genes were up-regulated more than five-fold, and 8 genes (CCL4, IL1β, IL1α, CCL2, ISG15, CCL8, LOC780407, CXCL2) up-regulated more than 10-fold.

**Table 2:** The DE genes associated with immune and inflammatory responses at 6 hpi.

SEQ_ID	p-value	FC Change	GeneName	description
NM_213779	0.000298	52.12223	CCL4	Sus scrofa chemokine (C-C motif) ligand 4 (CCL4), mRNA.
NM_214055	0.000173	41.07524	IL1B	Sus scrofa interleukin 1, beta (IL1B), mRNA.
NM_214029	0.001527	20.72427	IL1A	Sus scrofa interleukin 1, alpha (IL1A), mRNA.
NM_214214	0.000228	13.97029	CCL2	Sus scrofa chemokine (C-C motif) ligand 2 (CCL2), mRNA.
NM_001128469	0.0000129	13.800437	ISG15	Sus scrofa ISG15 ubiquitin-like modifier (ISG15), mRNA.
NM_001164515	0.000172	12.3193	CCL8	Sus scrofa chemokine (C-C motif) ligand 8 (CCL8), mRNA.
NM_001161434	7.71E-05	10.32473	LOC780407	Sus scrofa chemokine ligand 24-like protein (LOC780407), mRNA.
NM_001001861	0.000775	10.01722	CXCL2	Sus scrofa chemokine (C-X-C motif) ligand 2 (CXCL2), mRNA.
NM_001146128	0.008214	8.429539	IL7R	Sus scrofa interleukin 7 receptor (IL7R), mRNA.
ENSSSCT0000 0013077	0.000562	7.193156	NFKBIZ	Sus scrofa chromosome 13 Sscrofa10.2 partial sequence 165990981..166988044 reannotated via EnsEMBL
NM_214084	0.003889	7.036763	VEGFA	Sus scrofa vascular endothelial growth factor A (VEGFA), mRNA.
NM_214022	0.00066	6.881882	TNF	Sus scrofa tumor necrosis factor (TNF), mRNA.
XM_001929223	6.41E-05	6.076357	PNP	PREDICTED: Sus scrofa purine nucleoside phosphorylase (PNP), mRNA.
NM_214061	0.000237	5.901434	MX1	Sus scrofa myxovirus (influenza virus) resistance 1, interferon-inducible protein p78 (mouse) (MX1), mRNA.
NM_001008691	0.007205	5.879376	CXCL10	Sus scrofa chemokine (C-X-C motif) ligand 10 (CXCL10), mRNA.
NM_001100194	0.011522	5.642215	IFIH1	Sus scrofa interferon induced with helicase C domain 1 (IFIH1), mRNA.
NM_001160271	1.18E-05	5.595223	S100A8	Sus scrofa S100 calcium binding protein A8 (S100A8), mRNA.
XM_001925952	0.002127	4.301922	IFIT5	PREDICTED: Sus scrofa interferon-induced protein with tetratricopeptide repeats 5 (IFIT5), mRNA.
NM_213867	0.000619	4.162192	IL8	Sus scrofa interleukin 8 (IL8), mRNA.
NM_001164511	0.000316	4.145637	BCL2A1	Sus scrofa BCL2-related protein A1 (BCL2A1), mRNA.
NM_001048232	0.000114	4.033666	NFKB1	Sus scrofa nuclear factor of kappa light polypeptide gene enhancer in B-cells 1 (NFKB1), mRNA.
NM_001044552	5.65E-05	3.686546	LOC733 603	Sus scrofa serum amyloid A2 (LOC733603), mRNA.
NM_214303	4.32E-06	3.381383	OAS1	Sus scrofa 2′-5′-oligoadenylate synthetase 1, 40/46kDa (OAS1), mRNA.
XM_003127915	0.009899	3.04153	IFI44	PREDICTED: Sus scrofa interferon-induced protein 44 (IFI44), mRNA.
NM_001031796	0.000513	2.980078	OAS2	Sus scrofa 2′-5′-oligoadenylate synthetase 2, 69/71kDa (OAS2), mRNA.
XM_001924787	0.00041	2.957249	LOC1001 57000	PREDICTED: Sus scrofa chromosome 6 open reading frame 4, transcript variant 1 (LOC100157000), mRNA.
NM_001097416	0.012537	2.864776	MX2	Sus scrofa myxovirus (influenza virus) resistance 2 (mouse) (MX2), mRNA.
NM_001097445	0.000273	2.848918	CD14	Sus scrofa CD14 molecule (CD14), mRNA.
NM_001005150	2.73E-05	2.829609	NFKBIA	Sus scrofa nuclear factor of kappa light polypeptide gene enhancer in B-cells inhibitor, alpha (NFKBIA), mRNA.
NM_001177906	0.001647	2.748172	S100A9	Sus scrofa S100 calcium binding protein A9 (S100A9), mRNA.
NM_214379	0.006056	2.74367	PPARG	Sus scrofa peroxisome proliferator-activated receptor gamma (PPARG), mRNA.
NM_001244354	0.026452	2.451839	SEC61A1	Sus scrofa Sec61 alpha 1 subunit (S. cerevisiae) (SEC61A1), mRNA.
NM_001033011	0.008059	2.327992	FCGR1A	Sus scrofa Fc fragment of IgG, high affinity Ia, receptor (CD64) (FCGR1A), mRNA.
NM_001243435	0.025967	2.307106	ADORA3	Sus scrofa adenosine A3 receptor (ADORA3), transcript variant 1, mRNA.
NM_213761	0.016065	2.221215	TLR2	Sus scrofa toll-like receptor 2 (TLR2), mRNA.
AM177151	0.03376	2.168304	LOC1001 25542	Sus scrofa partial VDJ heavy chain gene for immunoglobulin heavy chain variable region, clone Pig3 CD5+ C13.
NM_214194	0.004432	2.082431	CD40	Sus scrofa CD40 molecule, TNF receptor superfamily member 5 (CD40), mRNA.
XM_003122031	0.001052	2.061163	TRIM14	PREDICTED: Sus scrofa tripartite motif containing 14 (TRIM14), mRNA.
NM_001078667	0.01484	2.051649	PSEN1	Sus scrofa presenilin 1 (PSEN1), mRNA.
AM177169	0.034076	2.033368	LOC100 125541	Sus scrofa partial VDJ heavy chain gene for immunoglobulin heavy chain variable region, clone Pig3 CD5- F12.
NM_001160272	6.64E-05	2.027732	S100A12	Sus scrofa S100 calcium binding protein A12 (S100A12), mRNA.
AF248289	0.048925	2.001558	LOC100 037926	Sus scrofa clone 3 immunoglobulin heavy chain mRNA, partial cds.

In addition to the functional groups observed at 6 hpi (namely inflammatory response, immune response, apoptosis, programmed cell death, signal transduction and so forth), the following functional groups of DE genes were observed at 15 hpi: cell-cell adhesion (e.g. CD274, CCN2, CLDN7, CD2), protein ubiquitination (e.g. MAPK9, PSMB8, PSMD10, PSMA5, ISG15), T cell proliferation, protein transport and so forth (Figure 1D). Similarly, a greater number of genes associated with immune and inflammatory responses were found at 15 hpi (Table S1).

### Pathway analysis

To gain insights into the different biological processes associated with *M. hyopneumoniae* infections at different times post-infection, pathway mapping of DE genes according to the Kyoto Encyclopedia of Genes and Genomes (KEGG) pathway database was performed. At 6 hpi, the predominant pathways included the cytokine-cytokine receptor interaction, chemokine signaling pathway, RIG-I-like receptor signaling pathway, Toll-like receptor signaling pathway, nucleotide-binding oligomerization domains (Nod)-like receptor (NLR) signaling pathway, proteasome, apoptosis signaling pathway, Cell adhesion molecules, Jak-STAT signaling pathway and PPAR signaling pathway. These results suggest that at an early stage of infection, the host initiated different strategies to activate immune and inflammatory responses to prevent infection (Table 3).

**Table 3:** DE genes analysis base on KEGG at 6 hpi.

Pathway Name	Number	Gene
Cytokine-cytokine receptor interaction	15	CCL2, CCL3L1, CCL4, CD40, CXCL10, CXCR4, IFNAR2, IL10RB, IL18, IL1A, IL1B, IL1R2, IL7R, IL8, TNF
Chemokine signaling pathway	13	CCL2, CCL3L1, CCL4, CCL8, CRK, CXCL10, CXCL2, CXCL2, CXCR4, IL8, NFKB1, NFKBIA, SRC
RIG-I-like receptor signaling pathway	11	CASP10, CXCL10, DHX58, IFIH1, IL8, IRF3, ISG15, NFKB1, NFKBIA, TANK, TNF
Toll-like receptor signaling pathway	11	CD14, CD40, CXCL10, IFNAR2, IL1B, IL8, IRF3, NFKB1, NFKBIA, TLR2, TNF
NOD-like receptor signaling pathway	8	CCL2, IL18, IL1B, IL8, NFKB1, NFKBIA, TNF, TNFAIP3
Phagosome	8	ATP6V1B2, C1R, CALR, CD14, FCGR1A, OLR1, SEC61A1, TLR2
Apoptosis	7	BIRC3, CASP10, IL1A, IL1B, NFKB1, NFKBIA, TNF
Cell adhesion molecules	7	CD274, CD40, CLDN4, CLDN7, ITGB8, SDC4, VCAM1
Jak-STAT signaling pathway	7	IFNAR2, IL10RB,IL7R, MYC, PIAS2, SOCS1, SOCS7
PPAR signaling pathway	6	ACSL1, ACSL4, ACSL5, GK, OLR1, PPARG

At 15 hpi, the phagosome, antigen processing and presentation, protein processing in endoplasmic reticulum became the most significant pathways. Other dominant pathways at late stages of infection included phagosome, RIG-I-like receptor signaling pathway, PPAR signaling pathway gap junction and tight junction (Table S2).

### STRING analysis of the relationships between DE genes

**Figure 2:**
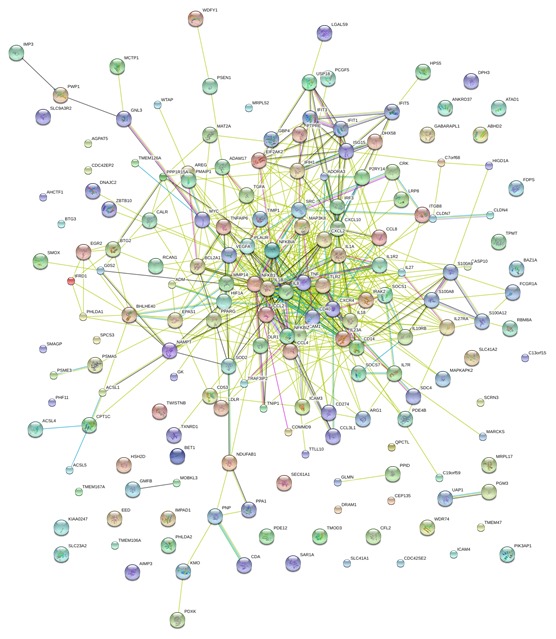
STRING analysis of the relationship between DE genes. The DE genes in PAM cells infected *M. hyopneumoniae* were analyzed using the STRING database. The network nodes represent the proteins encoded by the DE genes. Seven different colored lines link a number of nodes and represent seven types of evidence used in predicting associations. A red line indicates the presence of fusion evidence; a green line represents neighborhood evidence; a blue line represents coocurrence evidence; a purple line represents experimental evidence; a yellow line represents textmining evidence; a light blue line represents database evidence and a black line represents co-expression evidence.

DE genes were analyzed using STRING for predicting network of proteins encoded by DE genes [16], [17]. Predictions of functional association networks for all DE genes encoded proteins at 6 hpi are presented in Figure 2. The results indicated that genes MAP3KB, NFKB1, TNF, IL-1β, IL-8, TLR2, IKBA, BCL2L1, CD14, CXCR4, CXCL10 and IL-1R2 are associated according to experimental evidence, with involvement in many signaling pathways and other immune responses. The IL-1β, TLR2, PLK2, TNF-α, IKBA, IL-18 and TANK genes are involved in the NF-kappa-B pathway and in other immune responses. According to the STRING analysis a number of proteins (e.g. IL-1β, NFKB1, TLR2, IRF3, IL-7R, S100A8, BCL2A1, and ISG15) are integral molecules, linking to other proteins. However, many proteins failed to link to other proteins, and as such their functions were unrelated or unknown.

Based on database evidence, inflammatory cytokines IL-1β, IL-8 and TNF-α are the central genes of these protein interaction networks. According to text mining data, more than 60 DE genes were associated with inflammatory cytokines, including MYD88, CD14, AKT1, IRF7, IRF3, CCL4, CCL8, CCL2, CXCL2, CXCL10, BCL2A1, PPARG, CD40, ADORA3 and so on.

### Expression analyses of chemokines

**Figure 3:**
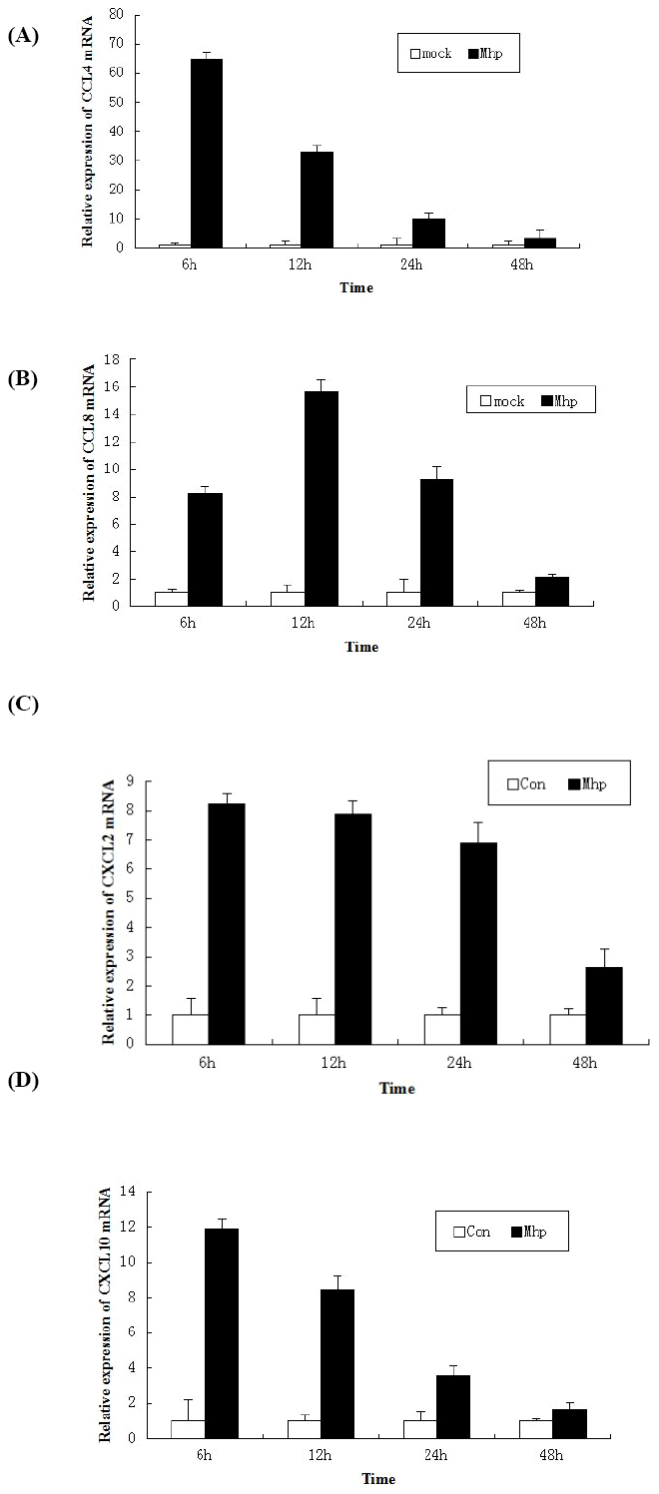
Expression kinetics of chemokines in PAM. PAMs were mock-infected, infected with *M. hyopneumoniae*. Cells were collected at the indicated time points, and subjected to real-time PCR to analyze the expression of CCL4 (A), CCL8 (B), CXCL2 (C) and CXCL10 (D).

When compared with the mock-inoculated PAMs, *M. hyopneumoniae*-infected PAMs inducted significantly higher levels of the chemokines from the transcription analysis data (Table 2). To understand the pattern of some chemokines expression in PAMs infected with *M. hyopneumoniae*, RNAs were extracted at different times post inoculation from the infected group and controls, and subjected to qRT-PCR analysis. The results showed that PAMs infected with *M. hyopneumoniae* exhibited significantly increased expression of CCL4, CXCL2 and CXCL10 mRNA at 6 h post-infection, and decreased at a steady-state level, with maximal production at 6 h post-infection. The CCL8 exhibited significantly increased expression at 6–24 h post-infection, and the maximal production was at 12 h post-infection (Figure 3).

## Discussion

*M. hyopneumoniae* is the primary cause of a chronic respiratory disease in pigs. And Mycoplasma infection correlates with the infection of other secondary respiratory pathogens by inducing the immunomodulation of host animals [18]–[21]. However, the mechanisms of immunomodulation are elusive. The previous study reported that the lung tissue injury about *M. hyopneumoniae* infections appear to be mainly caused by the host response [22]. And it indicated that the expression of proinflammatory cytokines were increased in the lung of *M. hyopneumoniae*-infected pigs [4]–[9]. These studies also suggested that the activation of host immune system played an important role in the pathogenesis and immune mechanisms for *M. hyopneumoniae* infection. Despite the fact that the current vaccine strategy can efficiently control *M. hyopneumoniae* infection, immunization failures exist in the field, and the molecular mechanisms underlying PRDC caused by *M. hyopneumoniae* remain largely unknown [23]. The present study aimed to identify genes involved in the immune response against *M. hyopneumoniae* in primary alveolar macrophages. Furthermore, by generating a comprehensive transcriptomic profile of the temporal *M. hyopneumoniae* pathogenic process in the host cells, we hoped to gain insights into the underlying molecular interactions and signaling pathways in the *M. hyopneumoniae* infection process.

This study is the first to report the use of GeneChip Porcine Genome Array for the investigation of transcriptional responses to *M. hyopneumoniae* infection. We found that more than 2000 transcripts with significant differential expression were produced in response to *M. hyopneumoniae* infections of PAM (Figure 1). Results from Gene Ontology, KEGG pathway and STRING analysis suggested that these DE genes belonged to a variety of functional categories and signal pathways.

Analysis of the expression of porcine genes after infection with *M. hyopneumoniae* showed that a large set of DE genes were involved in the immune response. TLR signaling plays an essential role in the innate immune response. TLR2 (up-regulated 2.22-fold) belongs to the TLR family and is expressed most abundantly in PAM (Table 2). TLR2 mediates the host response to mycoplasmal lipoproteins and has a fundamental role in pathogen recognition and activation of innate immunity. NF-κB (up-regulated 7.19 fold) is a family of inducible transcription factors involving pathogen- or cytokine-induced immune and inflammatory responses as well as cell proliferation and survival [24], [25]. The previously study indicated that *M. hyopneumoniae* infection could induce pro-inflammatory cytokines by the activation of the NF-κB [26]. These studies implied that *M. hyopneumoniae* might have developed sophisticated strategies for activation or inhibition of NF-κB pathway in order to survive in host cells. Further investigation to evaluate the exact mechanisms of *M. hyopneumoniae* in modulating NF-κB pathway will be required. Another receptor for bacteria, CD14 (up-regulated 2.85-fold) cooperates with TLR4 (which also recognizes lipopolysaccharides) via MYD88, leading to inflammatory responses in mycoplasmal infections [27], [28]. The signal transducer MYD88 (up-regulated 2.1 fold) is an adapter for almost all TLR signaling pathways, and acts via interferon regulatory factor 7 (IRF7), leading to activation of NF-κB, cytokine secretion and the inflammatory response. It was similar to the reports, which suggested that the interaction of *M. arthritidis* mitogen with TLR2 and TLR4 might play an important role in disease outcomes by *M. arthritidis* [29].

Many DE genes in this study are involved in the inflammatory response, including CCL4, IL-1β, IL-1α, CCL2, CCL8, CXCL2, TNF-α, CXCL10, IL-8, S100A8, PPARG; (peroxisome proliferator-activated receptor gamma), CD40 and so on. Furthermore, eleven genes involved with the TLR signaling pathway, eleven with the RLR signaling pathway, eight with the NLR signaling pathway, and thirteen with the chemokine signaling pathway, were found to be regulated (Table 3). These results reflect the up-stream signal cascades that could lead to secretion of inflammatory cytokines and chemokines.

Cytokines and chemokines are central mediators during host-pathogen interactions, including the clearance of invading microorganisms, as well as the initiation, progression, and resolution of inflammation in response to various microbes. In this study, chemokines CCL4, CCL2, CCL8, CXCL2 and CXCL10 was up-regulated more than 5-fold. CCL4, also known as macrophage inflammatory protein-1β (MIP-1β) is a CC chemokine with specificity for CCR5 receptors. It is a chemoattractant for natural killer cells, monocytes and a variety of other immune cells [30]. Cytokine CCL2 (chemokine (C-C motif) ligand 2, up-regulated 26.14-fold) is thought to bind to chemokine receptors CCR2 and CCR4. Studies demonstrating the contribution of T cells to disease pathogenesis, suggest the recruitment of T cells by chemokines (CCL4 and CCL8) elicited at the site of infection would most likely contribute to the pathogenesis of mycoplasma disease [31]. CXCL2 and CXCL10 are chemokines of the CXC subfamily, and CXC chemokines are particularly significant for leukocyte infiltration in inflammatory diseases. The expression kinetics of some chemokines showed that PAMs infected with *M. hyopneumoniae* exhibited significantly increased expression of these chemokines mRNA at 6 h post-infection, and decreased at a steady-state level, with maximal production at 6 h post-infection (Fig. 3A, 3C and 3D). It suggested that *M. hyopneumoniae* induced an inflammatory response at the early time point (6 HPI) of infection.

IL-1β, IL-8 and TNF-α are acute-phase proinflammatory mediators that promote inflammation and induce fever, tissue destruction, and, in some cases, shock and death [32], they play an integral role in shaping the inflammatory response against pathogens. Our study showed that *M. hyopneumoniae* could induce IL-1β, IL-8 and TNF-α expression (Table 2) in PAMs at 6 hpi. These results are in good agreement with previous study [4]–[9]. These results suggested that *M. hyopneumoniae* infection modulates the immune response of pigs by inducing several cytokines, and promotes the inflammatory response. Then it maybe cause immunosuppression to infected pigs.

Cell adhesion molecules (CAMs) perform various functions in fundamental cellular processes, including polarization, movement, proliferation and survival [33]. We discovered several DE genes that were related to cell adhesion during *M. hyopneumoniae* infection, including CD274, CLDN4, CLDN7, ITGB8, SDC4 and VCAM1. CD274 (up-regulated 4.11-fold) also known as programmed cell death 1 ligand 1 (PD-L1), which has been speculated to play a major role in suppressing the immune system during particular events such as pregnancy, tissue allografts, autoimmune disease and other disease states [34]. The VCAM-1(up-regulated 2.68-fold) also known as vascular cell adhesion molecule 1 or cluster of differentiation 106 (CD106), mediates the adhesion of lymphocytes, monocytes, eosinophils, and basophils to vascularendothelium. It also functions in leukocyte-endothelial cell signal transduction [35]. These cell adhesion molecules maybe play a important role in pathogenicity of *M. hyopneumoniae.*

Apoptosis plays an essential role in the development and maintenance of homeostasis in multicellular organisms. Moreover, apoptosis plays a crucial role in the pathogenesis of a number of infections [36]. Apoptosis is often considered as an innate defense mechanism that limits pathogen infection by eliminating infected cells. Bai et al indicated that the LAMP derived from *M. hyopneumoniae* induced apoptosis in porcine alveolar macrophage cell line through enhancing the production of NO, superoxide burst, and activation of caspase-3 [37]. In this study, 34 of the DE genes identified were known to be involved in apoptosis after *M. hyopneumoniae* infection. Such as, the caspase-10, TIMP1 (tissue inhibitor of metalloproteinases 1), BCL2-related protein A1, and PMAIP1 (Phorbol-12-myristate-13-acetate-induced protein 1) were up-regulated at 6 hpi to various degrees. Furthermore, seven genes were involved in the apoptosis signaling pathway (Table 3). BCL2-related protein A1 belongs to the Bcl-2 protein family, and is considered a pivotal player in apoptosis, especially for mitochondria-mediated apoptosis. Expression of PMAIP1 is regulated by the tumor suppressor p53, and PMAIP1 has been shown to be involved in p53-mediated apoptosis [38]. The glycoprotein TIMP1 is expressed from several tissues of biological organisms, is able to promote cell proliferation in a wide range of cell types, and may also possess an anti-apoptotic function [39]. Not surprisingly, pathogens target these proteins to induce or inhibit apoptosis. These findings were beneficial to research the mechanisms about *M. hyopneumoniae* infection induces apoptosis, and will contribute to a greater understanding of pathogenesis.

In summary, this is the first study to evaluate the gene expression profile of *M. hyopneumoniae* infected PAMs *in vitro*. Microarray analysis showed that the expressions of more than 2000 genes were altered after *M. hyopneumoniae* infection. These DE genes were involved in the inflammatory response, innate immune response, apoptosis, defense response, signal transduction, cell adhesion and so on. The data generated from the analysis of the overall pattern of innate immune signaling and proinflammatory, apoptotic and cell adhesion pathways activated by *M. hyopneumoniae* infection, could be used to screen potential host agents for reducing prevalence of *M. hyopneumoniae* infection, and to develop a greater understanding of the pathogenesis of *M. hyopneumoniae*.

## Materials and Methods

### Ethics

All pigs were purchased from the farm of Jiangsu Center of Disease Control, Nanjing, China. The healthy pigs (serologically negative for PRRSV, *M*. *hyopneumoniae* and porcine circovirus type 2) were sacrificed by the bloodletting after anesthesia under ethical approval granted by the Jiangsu Academy of Agricultural Sciences. The protocol was approved by the Science and Technology Agency of Jiangsu Province. The approval ID is NKYVET 2012-0035, granted by the Jiangsu Academy of Agricultural Sciences Experimental Animal ethics committee. All efforts were made to minimize animal's suffering. The PAMs were obtained from the sacrificed pigs under ethical approval for the purposes of research. The diseased piglets were not sacrificed.

### *M. hyopneumoniae* and cells

*M. hyopneumoniae* field strain XLW-1 was isolated from diseased pigs in Jiangsu Province, China. Porcine alveolar macrophages (PAMs) obtained from three 4–5-week-old *M. hyopneumoniae*-negative piglets, and serologically negative for PRRSV and porcine circovirus type 2 (PCV2), were prepared as described previously [14], [15]. Prior to infection, PAMs were mixed and confirmed negative for *M. hyopneumoniae*, PRRSV, PCV2, pseudorabies virus, and classical swine fever virus by PCR and RT-PCR. RPMI 1640 medium and fetal bovine serum (FBS) were obtained from GIBCO (Invitrogen). The isolated cells were grown and maintained in RPMI 1640 medium containing 10% (v/v) FBS at 37°C with 5% CO_2_.

In order to simulate natural conditions of *M. hyopneumoniae* infection, the tracheal ring were infected first. Tracheas were collected as previously described [40]. Briefly, the tracheas were excised aseptically from pigs and submerged in chilled PBS. Tracheas were washed with PBS, and transverse sections (approximately 0.5 cm thick) were prepared by making an incision between the tracheal rings. Each tracheal ring was placed in a 30-mm culture plate insert (Millipore, Bedford, Mass.) containing 3 ml of complete medium.

### Experimental design

The controls included uninfected PAMs and tracheas. *M. hyopneumoniae* (10^8^ CCU/ml) was inoculated onto the tracheal rings 10 h prior to their addition to PAMs (approximately 10^7^ cells). The *M. hyopneumoniae* group included the *M. hyopneumoniae*-infected tracheal ring, and the corresponding supernatant was transferred to culture PAMs. The mixture incubated at 37°C with 5% CO_2_. PAMs were harvested from the vessels' surface with a cell scraper at 6 and 15 hpi, and stored at −80°C until use.

### RNA preparation

Total RNA were extracted from PAM of each groups with Trizol (Invitrogen) then quantified using the Nano-Drop 1000 Spectrophotometer (Thermo Fisher Scientific Inc., USA). The quality of the RNA was checked by formaldehyde denaturing gel electrophoresis in 1.2% agarose gels, which showed dispersed bands (28S and 18S) without any obvious smearing patterns that would indicate degradation.

### Microarray hybridization

The RNA samples were sent to KangChen Bio-tech, China, for microarray hybridization. Each RNA sample from different PAM treatments was hybridized to one Roche NimbleGen Porcine Genome Expression Array (Roche). Briefly, double-stranded cDNA was synthesized from 6 mg of total RNA using a T7-oligo (dT) primer. The cDNA was further purified and converted into cRNA using an in vitro transcription reaction. Five *m*g cRNA was reverse transcribed to cDNA, fragmented, and then labeled with Cy3-dCTP (GE Healthcare) using Klenow. These labeled cDNA fragments were hybridized to NimbleGen Porcine Genome Expression Arrays for 16 h at 42°C using the Roche NimbleGen Hybridization System. Afterwards, the GeneChips were washed, stained, and then scanned with a Roche-NimbleGen MS200. The Roche NimbleGen Porcine Genome Expression Array contains over 135,000 probe sets, representing 45,023 transcripts and variants of pig from the database of RefSeq, Unigene and TIGR.

### Microarray data analysis

Raw data and statistical analyses were performed with Feature Extraction software. Normalization was performed per chip (normalized to 50th percentile) and per gene (normalized to the median) respectively. A statistical analysis of variance (ANOVA) model was applied to the data and the significance was showed by accepting a false discovery rate (FDR) of 0.05. A further cut-off threshold was applied based on a fold change of 2.0 between infected and control cells. Then all the DE genes were performed for hierarchical cluster (Ver.3.0) and TreeView (Ver.1.1.1) analyses. Genes with significant similarities to the transcripts in nr database based on BLASTX searches were selected for GO analysis, performed by MAS 3.0 software which was based on DAVID database (CapitalBio, Beijing, China). Annotation results were obtained by inputting the list of gene symbol as identifier. The pathway analysis was done by using the MAS 3.0 software which was based on the Kyoto Encyclopedia of Genes and Genomes (KEGG) database (http://www.genome.jp/kegg/pathway.html). Differentially expressed (DE) genes in porcine PAM infected with WD or KO were analyzed using STRING (http://string-db.org/), a database of known and predicted protein interactions. The raw and processed data discussed in this publication have been deposited in NCBI's Gene Expression Omnibus and are accessible through GEO series accession number GPL17577.

### Quantitative RT-PCR analysis

Quantitative RT-PCR (qRT-PCR) was used to validate selected data from the microarray experiments, and to follow the expression of a subset of genes over time. For each group, total RNA was extracted from PAMs using Trizol (Invitrogen), and 5 µg included as template for first strand cDNA synthesis using the Superscript II cDNA amplification System (Invitrogen), according to the manufacturer's instructions. GAPDH was included as an endogenous control. The specific primers used in the qRT-PCR assays are listed in Table 1. qRT-PCR was performed in triplicate for all reactions using the SYBR green detection system and an ABI 7500 real-time PCR system (Applied Biosystems, Warrington, UK). Relative standard curves for target and endogenous control primer pairs were performed to verify comparable PCR efficiencies, and once established the comparative (2-delta-delta) Ct method was applied.

### Expression analyses of chemokines

Total RNA was extracted from PAMs harvested at different times post-inoculation from each infection groups. CCL4, CCL8, CXCL2, CXCL10 expression were measured using specific primers (Table 1) and qRT-PCR, as described above. GAPDH was used as the endogenous control.

### Statistical analysis of qRT-PCR data

Statistical analyses were carried out using Microsoft Excel 2007 (Microsoft Co., USA). Differences between groups were assessed by one-way repeated measures ANOVA, followed by Tukey's multiple comparison tests. *P-*values less than 0.05 were considered to be statistically significant.

## Supporting Information

Table S1 **The DE genes associated with immune and inflammatory responses at 15 hpi.**
(DOC)

Table S2 **DE genes analysis base on KEGG at 15 hpi.**
(DOC)
