# Supplementary material for: Transcription Analysis of the Porcine Alveolar Macrophage Response to Mycoplasma hyopneumoniae
Source: PLoS One. 2014 Aug 6;9(8):e101968. doi: 10.1371/journal.pone.0101968 (PMC4123846; doi:10.1371/journal.pone.0101968)
Supplement: Table S1 — The DE genes associated with immune and inflammatory responses at 15 hpi. (DOC) [file pone.0101968.s001.doc]

**Additional table 1. The DE genes associated with immune and inflammatory responses at 15 HPI.**

| **SEQ_ID** | **p-value** | **FCAbsolute** | **GeneName** | **description** |
| --- | --- | --- | --- | --- |
| AB205105 | 0.003825 | 8.969482 | IGG2B | Sus scrofa IgG mRNA for immunoglobulin G CH2 and CH3 chain, partial cds." |
| NM_001033011 | 0.032772 | 4.359762 | FCGR1A | Sus scrofa Fc fragment of IgG, high affinity Ia, receptor (CD64) (FCGR1A), mRNA." |
| NM_001143693 | 0.000515 | 10.16764 | GZMH | Sus scrofa granzyme H (cathepsin G-like 2, protein h-CCPX) (GZMH), mRNA." |
| NM_001143710 | 0.00181 | 7.569142 | GZMB | Sus scrofa granzyme B (granzyme 2, cytotoxic T-lymphocyte-associated serine esterase 1) (GZMB), mRNA." |
| NM_001198926 | 0.004691 | 4.851853 | GZMA | Sus scrofa granzyme A (granzyme 1, cytotoxic T-lymphocyte-associated serine esterase 3) (GZMA), mRNA." |
| NM_214063 | 0.001143 | 3.4366 | HMGB2 | Sus scrofa high mobility group box 2 (HMGB2), mRNA." |
| NM_001044564 | 0.001242 | 3.539334 | SFN | Sus scrofa stratifin (SFN), mRNA." |
| NM_001044581 | 0.040681 | 3.506229 | TAP1 | Sus scrofa transporter 1, ATP-binding cassette, sub-family B (MDR/TAP) (TAP1), mRNA." |
| NM_001007518 | 0.029162 | 2.174197 | Hsp27 | Sus scrofa heat shock 27kDa protein 1 (Hsp27), mRNA." |
| XM_003354171 | 0.009851 | 2.301154 | MAPK9 | PREDICTED: Sus scrofa mitogen-activated protein kinase 9, transcript variant 2 (MAPK9), mRNA." |
| NM_214319 | 0.013207 | 2.443703 | PKR | Sus scrofa double stranded RNA-dependent protein kinase (PKR), mRNA." |
| NM_001004027 | 0.008484 | 2.217393 | HMOX1 | Sus scrofa heme oxygenase (decycling) 1 (HMOX1), mRNA." |
| NM_001011727 | 0.009916 | 3.194682 | AHCY | Sus scrofa adenosylhomocysteinase (AHCY), mRNA." |
| NM_001243435 | 0.002998 | 5.380138 | ADORA3 | Sus scrofa adenosine A3 receptor (ADORA3), transcript variant 1, mRNA." |
| NM_001033013 | 0.012494 | 5.829165 | FCGR2B | Sus scrofa Fc fragment of IgG, low affinity IIb, receptor (CD32) (FCGR2B), mRNA." |
| NM_001244215 | 0.029333 | 4.938922 | C5AR1 | Sus scrofa complement component 5a receptor 1 (C5AR1), mRNA." |
| NM_001123134 | 0.014168 | 2.91773 | LIPA | Sus scrofa lipase A, lysosomal acid, cholesterol esterase (LIPA), mRNA." |
| NM_001099923 | 0.04204 | 2.082178 | MYD88 | Sus scrofa myeloid differentiation primary response gene (88) (MYD88), mRNA." |
| XM_001925952 | 0.026608 | 3.634888 | IFIT5 | PREDICTED: Sus scrofa interferon-induced protein with tetratricopeptide repeats 5 (IFIT5), mRNA." |
| NM_001100194 | 0.039899 | 2.234398 | IFIH1 | Sus scrofa interferon induced with helicase C domain 1 (IFIH1), mRNA." |
