# Supplementary material for: Transcription Analysis of the Porcine Alveolar Macrophage Response to Mycoplasma hyopneumoniae
Source: PLoS One. 2014 Aug 6;9(8):e101968. doi: 10.1371/journal.pone.0101968 (PMC4123846; doi:10.1371/journal.pone.0101968)
Supplement: Table S2 — DE genes analysis base on KEGG at 15 hpi. (DOC) [file pone.0101968.s002.doc]

**Additional table 2. DE genes analysis base on KEGG at 15 HPI**

| **Pathway Name** | **Number** | **Gene** |
| --- | --- | --- |
| Phagosome | 8 | C1R, CALR, FCGR1A, LAMP2, TAP1, TAP1, TAP2, TAP2 |
| Antigen processing and presentation | 7 | CALR, NFYA, PSME2, TAP1, TAP1, TAP2, TAP2 |
| Protein processing in endoplasmic reticulum | 7 | CALR, DDIT3, DNAJB1, MAPK9, PKR, TRAM1, UBE2J1 |
| Proteasome | 5 | PSMB10, PSMB8, PSMB8, PSMB9, PSME2 |
| RIG-I-like receptor signaling pathway | 4 | IFIH1, IRF7, ISG15, MAPK9 |
| PPAR signaling pathway | 4 | ANGPTL4, LPL, PPARG, SCD |
| Phagosome | 7 | ACTB, ACTB, FCGR2B, TUBA1B, TUBA4A, TUBB2A, TUBB2B |
| Gap junction | 6 | GBI1, GJA1, TUBA1B, TUBA4A, TUBB2A, TUBB2B |
| Tight junction | 4 | ACTB, ACTB, GBI1, HCLS1 |
